# A hospital-based birth defects surveillance system in Kampala, Uganda

**DOI:** 10.1186/s12884-019-2542-x

**Published:** 2019-10-22

**Authors:** Daniel Mumpe-Mwanja, Linda Barlow-Mosha, Dhelia Williamson, Diana Valencia, Robert Serunjogi, Ayoub Kakande, Joyce Namale-Matovu, Jolly Nankunda, Doreen Birabwa-Male, Margaret Achom Okwero, Jesca Nsungwa-Sabiiti, Philippa Musoke

**Affiliations:** 1 Makerere University - Johns Hopkins University Research Collaboration, Kampala, Uganda; 2 US Centers for Disease Control and Prevention (CDC), Atlanta, USA; 3 Medical Research Council/Uganda Virus Research Institute and London School of Hygiene & Tropical Medicine Uganda Research Unit, Entebbe, Uganda; 4 Makerere University College of Health Sciences, Kampala, Uganda; 5 Mulago National Referral Hospital, Kampala, Uganda; 6 US Centers for Disease Control and Prevention (CDC), Kampala, Uganda; 7 Community Health Department, Ministry of Health, Kampala, Uganda

**Keywords:** Birth defects, Congenital anomalies, Birth prevalence, Hospital-based surveillance, Uganda

## Abstract

**Background:**

In 2010, the World Health Assembly passed a resolution calling upon countries to prevent birth defects where possible. Though birth defects surveillance programs are an important source of information to guide implementation and evaluation of preventive interventions, many countries that shoulder the largest burden of birth defects do not have surveillance programs. This paper shares the results of a hospital-based birth defects surveillance program in Uganda which, can be adopted by similar resource-limited countries.

**Methods:**

All informative births, including live births, stillbirths and spontaneous abortions; regardless of gestational age, delivered at four selected hospitals in Kampala from August 2015 to December 2017 were examined for birth defects. Demographic data were obtained by midwives through maternal interviews and review of hospital patient notes and entered in an electronic data collection tool. Identified birth defects were confirmed through bedside examination by a physician and review of photographs and a narrative description by a birth defects expert. Informative births (live, still and spontaneous abortions) with a confirmed birth defect were included in the numerator, while the total informative births (live, still and spontaneous abortions) were included in the denominator to estimate the prevalence of birth defects per 10,000 births.

**Results:**

The overall prevalence of birth defects was 66.2/10,000 births (95% CI 60.5–72.5). The most prevalent birth defects (per 10,000 births) were: Hypospadias, 23.4/10,000 (95% CI 18.9–28.9); Talipes equinovarus, 14.0/10,000 (95% CI 11.5–17.1) and Neural tube defects, 10.3/10,000 (95% CI 8.2–13.0). The least prevalent were: Microcephaly, 1.6/10,000 (95% CI 0.9–2.8); Microtia and Anotia, 1.6/10,000 (95% CI 0.9–2.8) and Imperforate anus, 2.0/10,000 (95% CI 1.2–3.4).

**Conclusion:**

A hospital-based surveillance project with active case ascertainment can generate reliable epidemiologic data about birth defects prevalence and can inform prevention policies and service provision needs in low and middle-income countries.

## Background

Birth defects are structural or functional anomalies that occur during intrauterine life and can be identified prenatally, at birth or sometimes later in infancy. The causes of birth defects are not well known; about half can be linked to a specific cause [1, 2]. The known risk factors include genetic alterations and environmental influences, such as nutritional deficiencies and fetal infections [3]. Birth defects contribute to a significant proportion of infant and child mortality. Annually, over 8 million children (6%) of the total births worldwide are born with serious birth defects [2, 4]. In addition, at least 3.3 million children less than 5 years of age die annually because of serious birth defects [4]. The majority of children who survive with birth defects may be physically and mentally disabled for life [2, 4]. Resource-limited settings experience the largest burden of birth defects worldwide with 94% of all birth defects and 95% of the related deaths [3, 4]. The high burden in these countries is attributed to a combination of factors, which include a high fertility rate, nutritional deficiencies, exposure to teratogens, weak regulation of medication, and high prevalence of congenital infections [4–6].

Knowledge of birth defects epidemiology is important to guide implementation and evaluation of preventive interventions [4, 7, 8]. Birth defects surveillance programs are helpful in providing policy makers with ongoing reliable epidemiological information [9]. However, most resource-limited settings lack birth defects surveillance systems and therefore have unreliable epidemiological data [4, 6, 8]. In 2010, the 63^rd^ World Health Assembly passed a resolution calling upon countries to prevent birth defects wherever possible, to implement screening programs, and to provide ongoing support and care to children with birth defects and their families [10].

Uganda is a resource-limited country that lacks recent accurate data on the prevalence of birth defects and a national birth defects registry [4, 6]. The 2006 March of Dimes global report for birth defects estimated a birth defects prevalence of 60.9 per 1000 births in Uganda, with prevalence of neural tube defects being 1.3 per 1000 births [4]. Studies conducted in Uganda have shown widely varying birth defects prevalence rates. A hospital-based descriptive study conducted at Mulago hospital between 1956 and 1957 among 2068 live and stillbirths estimated a prevalence of 540/10,000 [11], while another descriptive cross-sectional study conducted over 4 months among 754 newborns at Mulago hospital estimated a prevalence of 440/10,000 live births [12]. However, a randomized controlled trial conducted in Entebbe, Uganda, to determine the effect of helminths and their treatment in pregnancy and in young children on immunologic and disease outcomes in childhood among 2345 newborns between 2003 and 2005 reported a prevalence of 203/10,000 births [13]. All these studies included small sample sizes and used different data collection methods and birth defects definitions.

In 2014, Makerere University – Johns Hopkins University Research Collaboration in collaboration with the United States Centers for Disease Control and Prevention (US-CDC) initiated a hospital-based birth defects surveillance system with a nested case-control study in Kampala, Uganda. The purpose of the surveillance was to provide an accurate estimate of the prevalence of major external birth defects from August 2015 to December 2017 at four hospitals in Kampala, Uganda. This paper shares the progress and results of this birth defects surveillance system. This may serve as a guide to other resource-limited countries that are interested in establishing birth defects surveillance systems that can generate comparable prevalence data and may generate knowledge on risk factors and potential interventions for birth defects prevention from the nested case-control study within the surveillance system.

## Methods

### Project design

The birth defects surveillance system uses active case ascertainment and includes obtaining demographic and basic medical information for all births delivered at the participating hospitals. All newborns are examined for major external birth defects by trained midwives. A woman who delivers a baby with a major external birth defect is asked if photographs can be taken of her newborn to help with the diagnosis of the birth defect. Written informed consent is obtained before photographs are taken. If a photograph is not possible, midwives draw and write a detailed description of the defect.

**Fig. 1:**
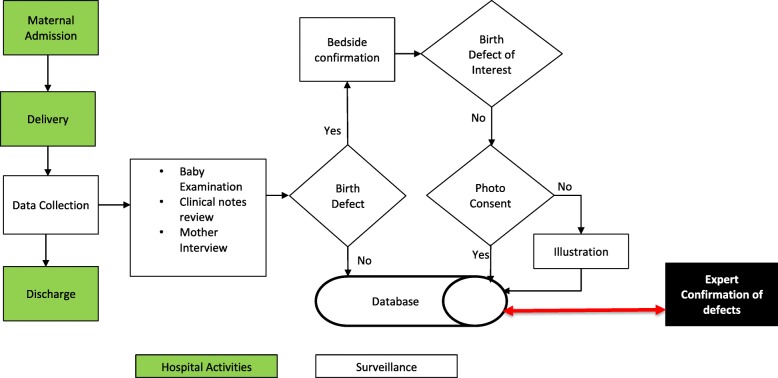
Surveillance system activity flow

Surveillance data are obtained from multiples sources, which include review of patient medical records, interviewer administered questionnaires, and newborn physical examination findings. Figure 1 illustrates the surveillance system activity flow.

All required ethics approvals were obtained as per the Uganda National Council for Science and technology (UNCST) guidelines [14]. This surveillance study was approved by the Joint Clinical Research Centre institutional review board/ethics committee and the US Centers for Disease Control and Prevention Institutional Review Board (IRB) (protocol # 6606.0). The surveillance was also approved by the Uganda National Council of Science and Technology (Ref: HS 1693),

### Study population

The surveillance system is being conducted at four hospitals, including one public/government hospital, Mulago National Referral Hospital and three faith-based private not-for-profit hospitals (Mengo hospital, St. Francis Hospital, Nsambya and Uganda Martyrs Hospital, Lubaga), in Kampala, Uganda. They were selected based on findings from a review of 2012 annual health data from the Ministry of Health, the 2012 annual hospital reports and the Uganda Demographic and Health Survey (UDHS) 2011. While the UDHS 2011 estimated 93% of births in Kampala were health-facility based, the Ministry of Health, 2012 annual health report and the 2012 annual hospital reports revealed 55% of the births in Kampala were at these four hospitals. The four hospitals included in this surveillance project have approximately 50,000 births annually, Mulago National Referral Hospital contributes 60.0% of births while Mengo Hospital, St. Francis Hospital, Nsambya and Uganda Martyrs Hospital, Lubaga contribute 12.0, 13.4 and 14.0% respectively. The time period for this surveillance project is approximately 4 years, during which we would expect to capture approximately 200,000 births. Assuming a birth defects prevalence range of 13.0 per 10,000 births for central nervous system defects to 87.0 per 10,000 births for musculoskeletal defects [4], we expect between 260 and 1740 newborns with each major external birth defect during this time period.

### Inclusion criteria

All informative births (live, stillbirths and spontaneous abortions), regardless of gestational age, at the four hospitals are included in the birth defects surveillance system. Informative births are those in which the newborn is well formed enough to ascertain the presence or absence of an external birth defect. Birth defects must be diagnosed at birth, during the newborn hospitalization period, or before discharge from the hospital. If prenatal diagnosis of birth defects is available, confirmation must be done at birth. In Uganda, elective termination of pregnancies is not legal except when it preserves maternal life and with consent by two registered physicians. However, all informative spontaneous abortions are included regardless of gestational age.

### Exclusion criteria

Births outside the four surveillance hospitals and un-informative macerated stillbirths are excluded.

### Newborn examination

All live births and stillbirths are examined by a trained surveillance midwife within 2 h of birth or as soon as feasible, without interrupting the first breastfeeding or preparation for burial. A systematic examination that is “head to toe” and “front to back” is used for every live birth and stillbirth to identify major external birth defects. During this examination, standard measurements are collected, including weight, head circumference and body length. Examination of all newborns is conducted by the surveillance midwife in the presence of the mother and/or relative where possible. All care of live newborns with birth defects is provided through routine care by the hospitals, which includes referral to specialists when available.

### Data collection

Data are collected by the surveillance midwife within 24 h after delivery using android-based tablets with paper forms as a back-up. All data collection forms were programmed using Open Data Kit (ODK), an open source data collection platform. All data entered on the tablet are encrypted and only completed forms are transcribed and transmitted to the main application server via internet. To ensure confidentiality, all tablets are password protected, and data are encrypted during transmission to protect it from unauthorized access. For every birth in the four participating hospitals, a surveillance form is completed by the midwife. Surveillance data include maternal demographic data such as age, tribe, address at the time of conception and current residence; brief maternal pregnancy history such as antenatal visit history, parity and history of previous birth defects; HIV sero-status and ART exposure; newborn characteristics such as sex, gestational age, anthropometric measures and presence or absence of birth defects; birth outcome such as live birth, stillbirth and spontaneous abortion. Each mother/newborn pair is assigned a unique study identification number that is generated automatically by the tablets. For multiple deliveries, a form is completed for each newborn.

### Ascertainment of birth defects

A mother who delivers a child with any major external birth defect is asked if photographs of her child can be taken. If she is willing, written informed consent is obtained and photographs are taken by the trained surveillance midwife using the Android-based tablet. Photographs are taken from several views, including a view of the entire fetus or newborn plus several focused views of the birth defect(s). Many major external birth defects are identified; however this surveillance focuses on birth defects of interest listed in Table 1.

**Table 1:** The birth defects of interest to the surveillance system

Birth defects category	Birth defects	ICD-10 RCPCH^a^ codes
Neural tube defects	Anencephaly	Q00.0
	Craniorachischisis	Q00.1
	Iniencephaly	Q00.2
	Encephalocele	Q01.0 – Q01.2, Q01.8 – Q01.9
	Spina bifida	Q05.0 – Q05.9
Congenital CNS^b^ malformations	Microcephaly	Q02
Congenital eye malformations	Microphthalmia and Anophthalmia	Q11 – Q11.1 and Q11.2
Congenital ear malformations	Microtia and Anotia	Q17.2 and Q16.0
Orofacial clefts	Cleft palate alone	Q35.1 – Q35.99, Q38.5,
	Cleft lip alone	Q36.0, Q36.99,
	Cleft lip with cleft palate	Q37.0 – Q37.99
Congenital absence, atresia and stenosis of the large intestine	Imperforate anus	Q42.2, Q42.3
Congenital malformations of the genital organs	Hypospadias	Q54.0 – Q54.3, Q54.8 – Q54.9
Congenital deformities of the feet	Talipes equinovarus	Q66.0
Limb reduction deficiencies	Limb reduction deficiencies	Q71.0 – Q73.8
Abdominal wall defects	Exomphalos/Omphalocele	Q79.2
	Gastroschisis	Q79.3

^a^10^th^ International Classification of Diseases modified by the Royal College of Paediatrics and Child Health adaptation

^b^CNS Central nervous system

In addition to taking photographs, the examining midwife writes a narrative description of the birth defect(s), detailing the location, size, appearance and other specific details necessary for an independent person who has not seen the infant to envision the birth defect(s) and make a diagnosis. The surveillance midwife then requests a study physician to do an independent bedside examination of any birth that she suspects has a birth defect. The study physician makes an independent diagnosis and prepares an additional independent narrative description. Both the surveillance midwife and the study physician assign diagnosis codes based on the 10^th^ International Classification of Diseases modified by the Royal College of Paediatrics and Child Health adaptation [15], which are either pre-programed in the tablet or available for reference on the tablet. The photographs and the two narrative descriptions are reviewed by the study review team (co-principal investigator and program manager), who may modify the narrative description, diagnosis and diagnostic code when necessary. The final decision is sent to CDC for confirmation of final diagnosis and code assignment.

In situations where mothers do not provide consent for photographs to be taken of their newborns, the surveillance midwives make illustrations of the birth defects, write detailed narrative descriptions of the birth defects, and photograph the illustrations.

The data collection software (ODK) links the photographs of the infant or the illustration with the mother’s surveillance information.

### Quality control, assurance and monitoring

Quality control and assurance are addressed in several ways. To standardize study activities, all study staff were trained on the principles of Good Clinical Practice [16], the study protocol, and how to conduct the surveillance activities. In addition, standard operating procedures were developed to ensure systematic collection of data and reduce interpersonal and inter-site variability.

Study data undergo three levels of quality control. Quality control level 1 combines use of real-time electronic and manual data checks. Quality control level 2 is a manual data check completed by study research assistants to ensure data completeness and validity. Quality control level 3 combines both manual and electronic reviews that are completed by the data managers, program manager and investigators.

To ensure inclusion of all births, hospital delivery registers are reconciled with the information in the database on a regular basis. Data from births that may have been discharged from the hospital and not included by surveillance midwives are abstracted from the patient medical file and entered by surveillance research assistants.

Quality assurance activities are regularly conducted by the program manager and CDC project monitors. The program manager regularly reviews 10.0% of randomly selected data collected in the past month to assess quality, completeness and regulatory compliance and implements corrective and preventive measures. These data include all newborns with birth defects, all cases and controls and a simple random sample of data from other participants not in the mentioned categories to add up to 10.0% data collected each month.

### Data analysis

Descriptive statistics of the population included in the surveillance system were generated, including maternal characteristics and infant characteristics. This includes the distribution of birth defects by maternal age, parity, maternal HIV sero-status, newborn sex, birth outcome, type of pregnancy, and mode of delivery.

The birth defects prevalence at the four surveillance hospitals was calculated for each major birth defect by aggregating the number of birth defect cases as the numerator and the total number of informative births (live births, stillbirths and spontaneous abortions) at the surveillance hospitals as the denominator. An infant or fetus with multiple birth defects was counted as a separate case for each defect [17]. Prevalence was expressed per 10,000 informative births (live births, stillbirths and spontaneous abortions) using the following formula during a specific time period:

$$ \frac{Number\kern0.5em of\kern0.5em birth\kern0.5em defects\kern0.5em among\kern0.5em live\kern0.5em birth s,\kern0.5em stillbirths\kern0.5em and\kern0.5em spontaneous\kern0.5em abortions}{Number\kern0.5em of\kern0.5em informative\kern0.5em live\kern0.5em birth s,\kern0.5em stillbirths\kern0.5em and\kern0.5em spontaneous\kern0.5em abortions}\times 10, 000 $$

The 95% confidence interval for each prevalence estimate was calculated using Wilson bounds [18].

## Results

### Inclusion of births in the surveillance

Implementation of the surveillance was initiated in a phased approach beginning at Mulago hospital in August 2015 and covered all the four hospitals by October 2016. A total 67,543 mothers and 70,063 eligible births were registered at the four hospitals between August 2015 the close of December 2017. Of the 70,063 registered births, 69,766 (99.6%) were included in the surveillance. The majority of surveillance birth inclusions, 59,000 (85.1%) were in Mulago National Referral Hospital, while 4139 (5.9%) were included in Mengo hospital, 4153 (6.0%) in St. Francis Hospital, Nsambya and 2072 (3.0%) in Uganda Martyrs Hospital, Lubaga. Table 2 summarizes the surveillance performance per year.

**Table 2:** Summary of inclusion of births in the surveillance

Year	Eligible births	Births included in Surveillance, n (%)	Births not included in surveillance, n (%)
2015	7146	7091 (99.2)	55 (0.8)
2016	25,009	24,917 (99.6)	92 (0.4)
2017	37,908	37,758 (99.6)	150 (0.4)
Total	70,063	69,766 (99.6)	297 (0.4)

### Maternal and newborn characteristics

The majority of mothers delivering at the hospitals were in the age stratum of 20–29 years old, 42,761 (63.3%), while those of age strata of 30–39 years were 16,550 (24.5%), the mothers younger than 20 years contributed 7366 (10.9%) births, while mothers older than 40 years contributed 866 (1.3%) births. Most of the mothers, 37,166 (55.0%) had carried 1–3 pregnancies; while 9343 (13.8%) carried more than 3 pregnancies and 21,034 (31.1%) were primipara. The HIV sero-prevalence of the mothers was 9.6% compared to 6.0% for the national average. There were 36,352 (52.1%) male births compared to 33,401 (47.9%) female births. Most of the births were live 66,793 (95.7%). Singletons comprised 64,988 (93.2%) of the births, and vaginal deliveries were 48,191 (69.1%). Table 3 summarizes the prevalence of the birth defects of interest in the surveillance population.

**Table 3:** Prevalence of all birth defects of interest by maternal and newborn demographic characteristics

Demographics	Frequency n (%)	Number with Birth defect	Birth defects prevalence per 10,000 births (95% CI)
Maternal age^a^			
< 20 years	7366 (10.9)	40	54.3 (39.9–73.9)
20–29 years	42,761 (63.3)	206	48.2 (42.0–55.2)
30–39 years	16,550 (24.5)	99	59.8 (49.2–72.8)
> 40 years	866 (1.3)	11	127.0 (71.1–226.0)
Parity^a^			
0	21,034 (31.1)	112	53.2 (44.3–64.0)
1–3	37,166 (55.0)	189	50.9 (44.1–58.6)
> 3	9343 (13.8)	55	58.9 (45.3–76.5)
Maternal HIV sero-status^a^			
Positive	6494 (9.6)	27	41.6 (28.6–60.4)
Negative	60,907 (90.2)	326	53.5 (48–59.6)
Unknown	142 (0.2)	3	211.3 (72.1–602.7)
Newborn sex^b, c^			
Male	36,352 (52.1)	248	68.2 (60.3–77.2)
Female	33,401 (47.9)	112	33.5 (27.9–40.3)
Birth outcome^b^			
Live	66,793 (95.7)	320	47.9 (42.9–53.4)
Still	2511 (3.6)	39	155.3 (113.8–211.6)
Spontaneous abortion	462 (0.7)	5	108.2 (46.3–250.8)
Type of pregnancy^b^			
Singleton	64,988 (93.2)	339	52.2 (46.9–58.0)
Multiple	4778 (6.8)	25	52.3 (35.5–77.1)
Mode of Delivery^b^			
Vaginal	48,191 (69.1)	247	51.3 (45.3–58.0)
Caesarean	21,575 (30.9)	117	54.2 (45.3–64.9)

^a^Number of mothers (*N* = 67,543)

^b^Number of newborns (*N* = 69,766)

^c^13 Births had indeterminate sex

There were 461 mothers who delivered babies with birth defects of interest, 161 (34.9%) of these did not give consent to take photographs of their babies; however illustrations and narrative descriptions were made by the midwives and reviewed for confirmation by the birth defects expert. There were only 4 (0.9%) babies with birth defects that the midwives were unable to examine, but patient medical files had sufficient information which was extracted by the research assistants and was reviewed by the birth defects expert for confirmation of the diagnosis.

### Prevalence of the birth defects of interest

The overall prevalence for the major external birth defects of interest to the surveillance was 66.2/10,000 births (95% CI 60.5–72.5). The prevalence of the birth defects/10,000 births is summarized in Table 4. The most prevalent birth defects (per 10,000 births) were: Hypospadias, 23.4/10,000 (95% CI 18.9–28.9); Talipes equinovarus 14.0/10,000 (95% CI 11.5–17.1) and Neural tube defects, 10.3/10,000 (95% CI 8.2–13.0). The least prevalent were: Microcephaly, 1.6/10,000 (95% CI 0.9–2.8); Microtia and Anotia, 1.6/10,000 (95% CI 0.9–2.8) and Imperforate anus 2.0/10,000 (95% CI 1.2–3.4).

**Table 4:** Prevalence of major external birth defects of interest

Birth defects category	Number Identified			Prevalence per 10,000 births (95%CI)^a^
	Isolated	Multiple^c^	Total	
Neural tube defects	32	40	72	10.3 (8.2–13.0)
Microcephaly	5	6	11	1.6 (0.9–2.8)
Microphthalmia and Anophthalmia	0	16	16	2.3 (1.4–3.7)
Microtia and Anotia	3	8	11	1.6 (0.9–2.8)
Cleft palate alone	2	12	14	2.0 (1.2–3.4)
Cleft lip with or without cleft palate	24	15	39	5.6 (4.1–7.6)
Imperforate anus	0	14	14	2.0 (1.2–3.4)
Hypospadias^b^	64	21	85	23.4 (18.9–28.9)
Talipes equinovarus	56	42	98	14.0 (11.5–17.1)
Limb reduction defects	9	39	48	6.9 (5.2–9.1)
Omphalocele	21	16	37	5.3 (3.8–7.3)
Gastroschisis	14	3	17	2.4 (1.5–3.9)
Total	230	232	462	66.2 (60.5–72.5)

^a^Number of newborns (*n* = 69,766)

^b^Represents male hypospadias, the denominator was male births, *n* = 36,352

^c^These were part of multiple defects in a newborn, sequences and known syndromes do not contribute to this number

## Discussion

In this hospital-based birth defects surveillance, we found a birth prevalence of selected major external birth defects of 66.2/10,000 births. Hypospadias, Talipes equinovarus, and Neural tube defects were the most common birth defects identified however the surveillance team was able to identify rare birth defects such as: Microcephaly, Microtia and Anotia.

We did not find population based data from birth defects surveillance systems in similar low income settings [4, 19] to compare with birth prevalence rates of the defects included in our surveillance. The birth prevalence of neural tube defects estimated by our study is similar to the March of Dimes modeled estimate for the Uganda population [4]; however, the birth prevalence estimates of the other birth defects categories are generally lower than the March of Dimes modeled estimates. This is likely because our surveillance is urban and hospital-based while the March of Dimes estimates are based on the entire Ugandan population [4]. The birth prevalence for talipes equinovarus estimated by our study is slightly higher than that estimated for the Africa region by a systematic review and meta-analysis [20]. This difference in findings could be because the majority of studies included in systematic review and meta-analysis studied only live births unlike our study which included live births, stillbirths and informative abortions.

A surveillance study conducted in four urban hospitals in Dar es Salaam, Tanzania estimated neural tube birth prevalence similar to that estimated by our study [21]. However other hospital-based studies conducted in Uganda estimated different birth prevalence rates in some categories than those of our study, for comparable birth defects [11–13]. The differences are likely due to the small number of births included in those studies which were likely not representative of the larger population [22], inclusion of minor anomalies, and the difference in data collection methods.

### Strengths

This surveillance uses established birth defects surveillance methodology designed by a collaborative effort of the World Health Organization, the National Center on Birth Defects and Developmental Disabilities from the US-CDC, and the International Clearing House for Birth Defects Surveillance and Research [23]. To estimate the prevalence of major external birth defects, we implemented a hospital-based surveillance system in Kampala, where more than 94% of the resident women deliver in a health facility, according to the UDHS, 2016 [24]. The large number of deliveries at the four hospitals included in the surveillance system, together with the wide scope of easily identifiable major external birth defects, will provide an ongoing accurate estimation of the birth defects prevalence compared to previous studies conducted in Uganda [11–13, 25].

Unlike other studies which only include live births [23], this surveillance included stillbirths, spontaneous abortions and live births which minimized selection bias especially since some structural birth defects commonly occur among stillbirths thereby giving more accurate birth prevalence estimates.

The surveillance system collects a large volume of data with many variables that may allow assessing maternal antiretroviral therapy (ART) use, folate deficiency and other risk factors for birth defects and other pregnancy outcomes. These data may be pooled with data from similar studies to supplement and provide additional evidence [26], such as safety of new ART regimens introduced before and during pregnancy. Surveillance data may also be used to guide the need, and measure the impact of implementing proven prevention strategies such as food fortification and supplementation of folic acid for the prevention of spina bifida and anencephaly [27].

Unlike previous studies that have abstracted data from medical records to estimate birth defects prevalence [25, 28–30] and have faced a range of challenges, including incomplete documentation, lack of details, and inaccurate coding [31], this surveillance project uses multiple data sources, including real-time entry of examination findings by trained midwives, abstraction of data from medical records, maternal interviews, bedside confirmation of birth defect findings by a physician, photographs and illustrations, and narrative descriptions. These data allow for birth defect expert review of the identified birth defects to further improve the diagnostic accuracy. The use of multiple data sources also provides an opportunity for data verification conducted by the surveillance team and external monitors, thereby ensuring good data quality.

The use of real-time electronic data capture using tablets reduces the human resource needs and cost of supplies for the surveillance. Real-time data submission allows for timely review and analysis. While this surveillance project has implemented an elaborate three tier level of quality control, a simpler quality control plan could be implemented in new surveillance projects and expanded as needed.

Furthermore, the surveillance is conducted by trained midwives and research assistants, who receive performance feedback, ongoing birth defects training and supervisory support. The midwives also provide 24 h coverage of all the birthing units within the surveillance hospitals. This approach helps to ensure completeness of data.

### Limitations

Though the surveillance system is set-up in hospitals that deliver a majority of births in Kampala, the prevalence estimates from this surveillance cannot be generalized to the country because all the hospitals are urban while most of the population lives in rural areas with higher fertility rates compared to the urban population. In addition, there are fewer facility-based deliveries in rural areas of Uganda compared to the urban areas [24]. This surveillance system is not designed to include birth defects diagnosed after discharge from the hospitals and does not conduct diagnostic imaging for limb reduction defects or internal birth defects, such as congenital heart defects, and testing for functional birth defects, such as sickle cell disease. Use of diagnostic imaging and testing may provide more accurate estimates of birth prevalence rates for major internal and functional birth defects. Lack of follow-up also limits the assessment of survival among newborns with birth defects.

## Conclusion

Implementation of a hospital-based surveillance system in low resource settings can provide reliable prevalence estimates and trends over time for major external birth defects and can be used to assess associated risk factors for birth defects, including newer ART regimens and other drugs used in early pregnancy and adverse birth outcomes, such as preterm birth, low birth weight, and stillbirth. Though the results from this surveillance system may be used to guide policy on prevention and care interventions for birth defects, the experience obtained should be built on to design a surveillance system that can collect data from non-institutional births and rural populations to guide design of policies tailored to both rural and urban populations. In addition, the surveillance project can serve as a platform for further studies and a pilot for expansion to rural health facilities, other national and international healthcare facilities using the existing infrastructure. The lessons learned during this surveillance may stimulate interest in birth defects surveillance and prevention and guide implementation of birth defects surveillance in Uganda and other resource-limited settings.

## Data Availability

Not applicable.

## Abbreviations

ART: Antiretroviral therapy
CDC: Centers for Diseases Control and Prevention;
HIV: Human immunodeficiency virus
LNMP: Last normal menstrual period
ODK: Open Data Kit
UDHS: Uganda Demographic and Health Survey
US: United States
